# Classification of Cerebral Lymphomas and Glioblastomas Featuring Luminance Distribution Analysis

**DOI:** 10.1155/2013/619658

**Published:** 2013-06-06

**Authors:** Toshihiko Yamasaki, Tsuhan Chen, Toshinori Hirai, Ryuji Murakami

**Affiliations:** ^1^School of Electrical and Computer Engineering, Cornell University, Phillips Hall, Ithaca, NY 14853-5401, USA; ^2^Department of Information and Communications Engineering, The University of Tokyo, 7-3-1 Hongo, Bunkyo-ku, Tokyo 113-8656, Japan; ^3^Department of Diagnostic Radiology, Kumamoto University, 1-1-1 Honjo, Kumamoto City, Kumamoto 860-8556, Japan; ^4^Department of Medical Imaging, Kumamoto University, 4-24-1 Kuhonji, Kumamoto City, Kumamoto 862-0976, Japan

## Abstract

Differentiating lymphomas and glioblastomas is important for proper treatment planning. A number of works have been proposed but there are still some problems. For example, many works depend on thresholding a single feature value, which is susceptible to noise. In other cases, experienced observers are required to extract the feature values or to provide some interactions with the system. Even if experts are involved, interobserver variance becomes another problem. In addition, most of the works use only one or a few slice(s) because 3D tumor segmentation is time consuming. In this paper, we propose a tumor classification system that analyzes the luminance distribution of the whole tumor region. Typical cases are classified by the luminance range thresholding and the apparent diffusion coefficients (ADC) thresholding. Nontypical cases are classified by a support vector machine (SVM). Most of the processing elements are semiautomatic. Therefore, even novice users can use the system easily and get the same results as experts. The experiments were conducted using 40 MRI datasets. The classification accuracy of the proposed method was 91.1% without the ADC thresholding and 95.4% with the ADC thresholding. On the other hand, the baseline method, the conventional ADC thresholding, yielded only 67.5% accuracy.

## 1. Introduction

The purpose of this study is to present an objective and accurate tumor classification system that considers the luminance distribution of the whole 3D tumor region. Differentiating lymphomas and glioblastoma by noninvasive ways is an important problem because they require different chemotherapy regimens. For example, if the tumor is highly suspected to be lymphomas, stereotactic biopsy is usually recommended to confirm the diagnosis. If the tumor is highly suspected to be glioblastoma, craniotomy would be chosen. Chemotherapy regimens are different for the two tumors, as well.

Therefore, a number of works have been proposed. For instance, Toh et al. [1] proposed the ADC thresholding and the ADC ratio thresholding. The distribution of the ADC values was also discussed in [2]. Makino et al. [3] proposed standard uptake value (SUV) thresholding. In [4], a relative regional cerebral blood volume ratio was proposed. Calli et al. [5], on the other hand, used perfusion and diffusion MR imaging and introduced four parameters to differentiate the tumors. In [6], histogram analysis of the normalized cerebral blood volume in enhancing and perienhancing lesions was presented. Note that [1–6] do not include any image processing. Certain feature values are extracted from input MRIs by hand by experts [1–5] or regions of interest were specified by experts to generate the histograms [6]. Tumors were simply classified by thresholding using predefined static threshold values in [1–6]. An image-processing-based system can be found in [7]. In [7], texture analysis using Gabor wavelet coefficient thresholding was proposed, but this technique also relied on simple thresholding. Thresholding feature values is sensitive to noise. It is very easy to find exceptional cases for such simple thresholding. In addition, extracting parameters subjectively by the observers induces an interobserver variance problem. For instance, the ADC values extracted by experts differ from observers to observers. From this point of view, even the learning-based approaches which employ a lot of subjective parameters such as [8] would not become a solution to the aforementioned thresholding-based approaches. In addition, the analysis is done by using only a single or a few slices of the MRIs in most cases because segmenting the whole tumor region needs labor-intensive user interactions and takes a lot of time. For more robust and accurate tumor classification, analysis of the whole tumor region would be desired. For this purpose, we proposed a system that employed luminance distribution learning using the whole tumor region [9]. In the system, the classification accuracy was up to 87%.

The method proposed in this work is (1) objective because the input from users is as small as possible, (2) accurate by the combination of the luminance distribution analysis of the whole tumor region and the two thresholding methods, and (3) semiautomatic facilitating novice users to use the system easily. The 3D tumor segmentation and its luminance distribution analysis within a reasonable processing time have been made possible by our previous fast segmentation algorithm [10]. As a result, even novice users can classify the tumors accurately.

The system without the ADC thresholding is free of interobserver variances and achieves 91.1% accuracy. When the subjectively measured ADC value is included, the classification accuracy can be improved up to 95.4%. The main concept of our proposed algorithm has already been presented in [11]. In this paper, more detailed analysis and comparison are conducted to show the validity of the proposed method.

The rest of this paper is organized as follows. In Section 2, the proposed algorithm is described in detail. The experimental results are demonstrated in Section 3, followed by concluding remarks in Section 4.

## 2. Proposed Algorithm

It is often reported that the morphological appearance of the tumor does not allow direct judgments. However, it is also often observed that the lymphomas tend to have flatter and lower luminance as compared to glioblastomas and glioblastomas are brighter on the edge and darker in the center [6]. Therefore, glioblastomas have wider dynamic range in their luminance value. We use these different characteristics in two ways: one for thresholding using the luminance dynamic range and the other for the luminance distribution learning using SVM.

**Figure 1:**
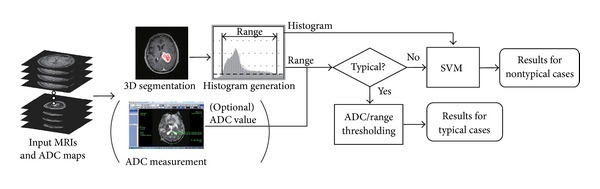
Flowchart of the proposed algorithm.

The flow chart of the proposed algorithm is shown in Figure 1. Firstly, the tumor region is segmented by using our fast 3D segmentation algorithm [10]. The required interaction is only tumor/nontumor seed setting, which does not require any expert knowledge. The processing time is about a few tens of seconds. Though the fast 3D tumor segmentation is not the scope of this paper, we would like to emphasize that the system was made reasonable because of the quick segmentation. In this stage, moderately accurate segmentation is enough because the segmentation results are used only for generating the luminance histograms, not for treatment design.

Then, the normalized histogram of the luminance of the tumor is generated automatically using the whole tumor region. In this paper, the voxel value range was quantized from 12 bits (4,096 levels) to 8 bits (256 levels) to make the histograms less sparse. By using the generated luminance histogram, the luminance range whose normalized frequency is larger than a predefined threshold (0.002 in our study) is detected.

At the same time, if experts are available, the average ADC value is measured. The region of interest (ROI), whose area is set about 50 mm^2^, is decided by the experts. This ADC value measurement is optional. 

According to [1], the ADC values tend to be smaller for lymphomas and large for glioblastomas. In the same manner, we found in our investigation that the luminance ranges are narrower for lymphomas and wider for glioblastomas. Although there are many exceptions as shown in our experiments, the tumors with very low ADC value can be regarded as lymphomas and those with very high ADC value can be regarded as glioblastomas. Therefore, we separate the typical cases and nontypical cases by using both the ADC value and the luminance range. Namely, we extract the four threshold values from the training data: the maximum ADC value and the maximum luminance range of the lymphomas (TH_ADC_lym_max⁡_ and TH_range_lym_max⁡_), and the minimum ADC value and the minimum luminance range of the glioblastomas (TH_ADC_gli_min⁡_ and TH_range_gli_min⁡_). There are two cases to consider.

**Figure 2:**
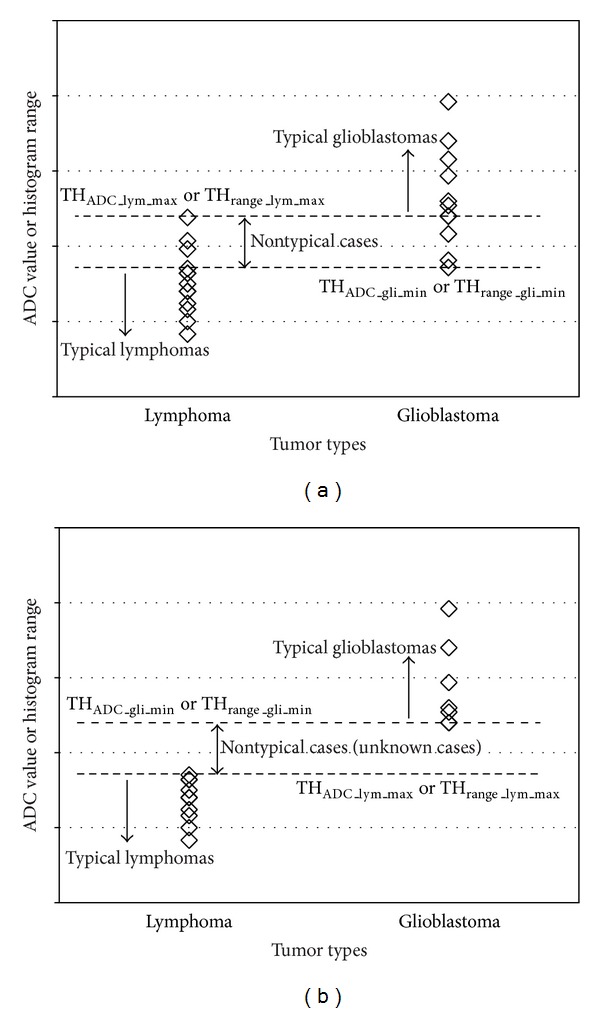
Concept of ADC and luminance range thresholding.

1.  TH_ADC_gli_min⁡_≤TH_ADC_lym_max⁡_ or  TH_range_gli_min⁡_≤TH_range_lym_max⁡_ (Figure 2(a)).

If the ADC value is smaller than TH_ADC_gli_min⁡_ or the luminance range is narrower than TH_range_gli_min⁡_, the tumor is regarded as a typical lymphoma. On the other hand, if the ADC value is larger than TH_ADC_lym_max⁡_ or the luminance range is wider than TH_range_lym_max⁡_, the tumor is regarded as a typical glioblastoma. Otherwise, the tumor is regarded as a nontypical case and classified by SVM, which is trained by the luminance histograms in the training dataset.

(2)  TH_ADC_gli_min⁡_ > TH_ADC_lym_max⁡_  and  TH_range_gli_min⁡_ > TH_range_lym_max⁡_ (Figure 2(b)).

If the ADC value is smaller than TH_ADC_lym_max⁡_ or the luminance range is narrower than TH_range_lym_max⁡_, the tumor is regarded as a typical lymphoma. On the other hand, if the ADC value is larger than TH_ADC_gli_min⁡_ or the luminance range is wider than TH_range_gli_min⁡_, the tumor is regarded as a typical glioblastoma. Those between TH_ADC_gli_min⁡_ and TH_ADC_lym_max⁡_ and between TH_range_gli_min⁡_ and TH_range_lym_max⁡_ are regarded as nontypical (unknown) cases and classified by SVM. This case rarely happens when the number of training data is large enough.

Note that the thresholding is used to extract typical cases. Therefore, the problem of thresholding-based method pointed out in Section 1 does not arise in this case. In addition, the four threshold values are decided automatically from the training dataset. Preliminary experiments to set proper threshold values are not needed. 

The advantage of the proposed system is that the ADC measurement can be omitted if experienced observers are not available. In this case, the required interaction from the users is only seed setting for the segmentation. The histogram generation, range thresholding, and classification using SVM can be done automatically. Therefore, anyone can reproduce the same results. Though a histogram-based approach was proposed in [6], the histograms were generated only from ROIs specified by the observers, not from the whole tumor region. And the classification was still based on thresholding a single parameter that was extracted from the histograms.

## 3. Experimental Results

### 3.1. Experimental Setup

**Figure 3:**
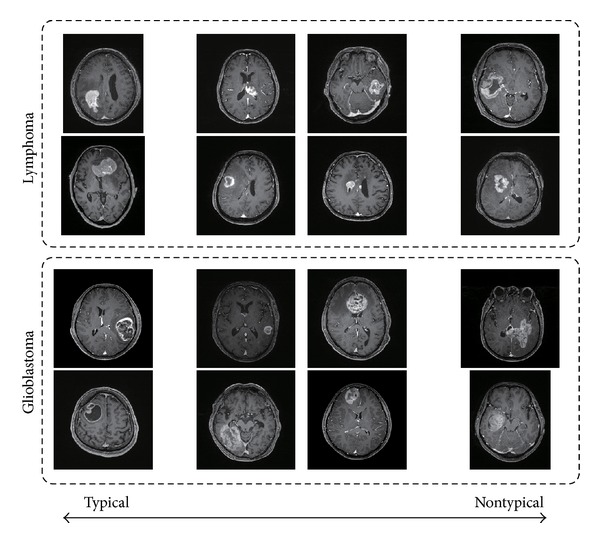
Sample MRIs of lymphomas and glioblastomas.

We retrospectively reviewed the MR images of 40 patients with histologically proved glioblastomas (*n* = 20) and lymphomas (*n* = 20). All tumors were pathologically proved. The resolutions of structural MRIs and ADC maps were 256 × 256 × 160 and 128 × 128 × 22, respectively. There were 22 male and 18 female patients, and their ages ranged from 12 to 91 years, with a mean of 65 years and a median of 69 years. Typical and nontypical cases are included in the dataset. The number of typical/nontypical cases is not listed here because it is hard to define typicalness/nontypicalness. Some example images are shown in Figure 3. As shown in the figure, some of the lymphomas and glioblastomas look very similar to each other and hard to differentiate just by looking at the MRIs. The ADC values were measured by a neuroradiologist with 19 years' experience (T. Hirai). A linear kernel [12] was used if not mentioned otherwise.

**Figure 4:**
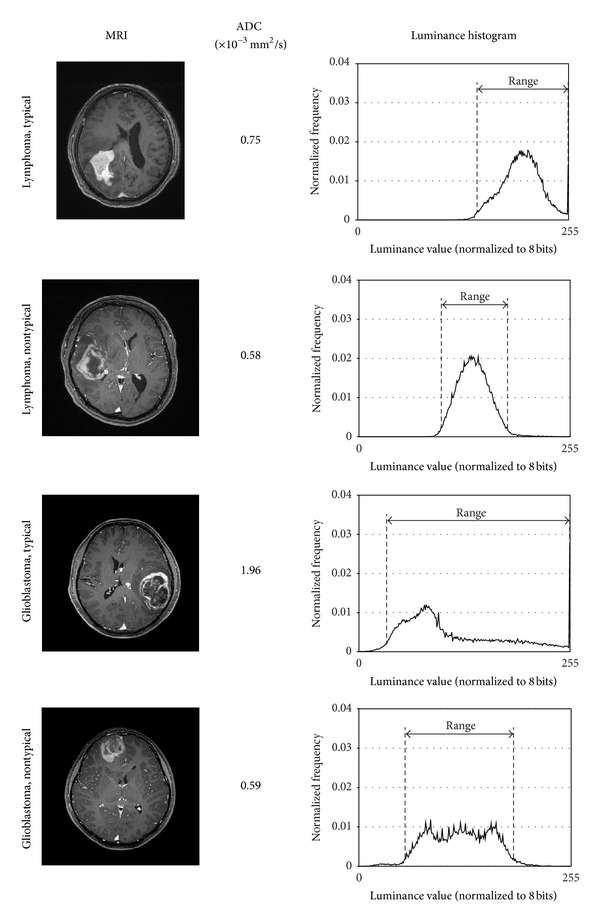
Sample images and extracted luminance histograms of typical/nontypical lymphomas/glioblastomas.

In Figure 4, typical and nontypical cases of lymphoma and glioblastoma and their luminance histograms are shown. As discussed in Section 3, the luminance histograms of lymphoma tend to have narrower luminance range and those of glioblastomas tend to have wider luminance range.

### 3.2. Classification Performance of Conventional Single Value Thresholding

**Figure 5:**
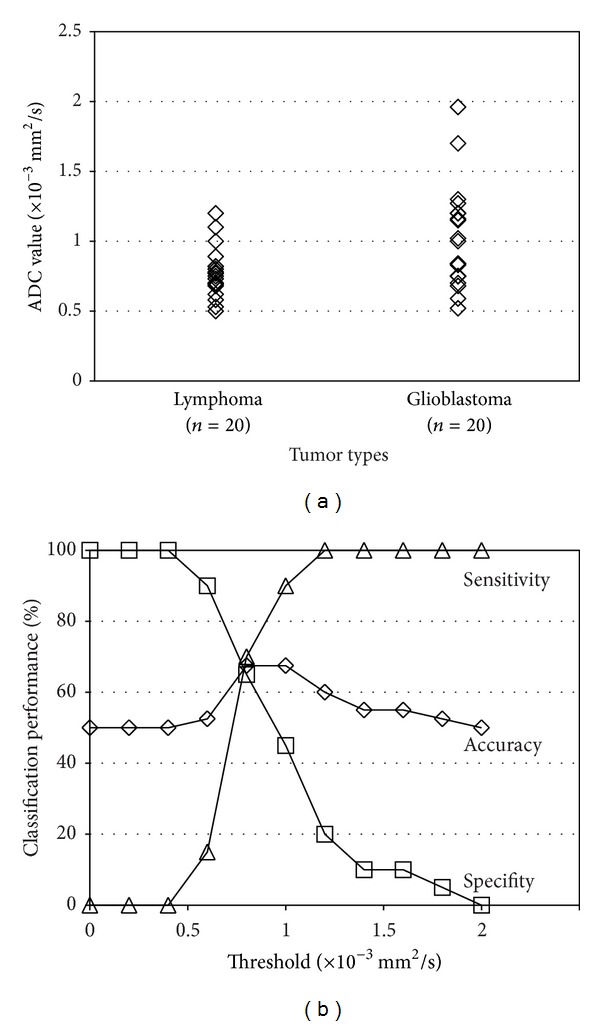
(a) Distribution of the ADC values. (b) Classification performance when ADC thresholding [1] is used. In the sensitivity/specifity calculation, lymphomas were considered as “positive” and glioblastomas were considered as “negative.”

Figure 5 demonstrates the distribution of the ADC values of lymphomas and glioblastomas (Figure 5(a)) and the differentiation performance as a function of the threshold value (Figure 5(b)). It is observed that the ADC values of the lymphoma and those of the glioblastomas overlap each other and therefore the ADC thresholding [1] does not work well. The best accuracy was only 67.5% when the threshold was 0.8–1.0 × 10^−3^ mm^2^/s, which is slightly better than the chance level (50%).

**Figure 6:**
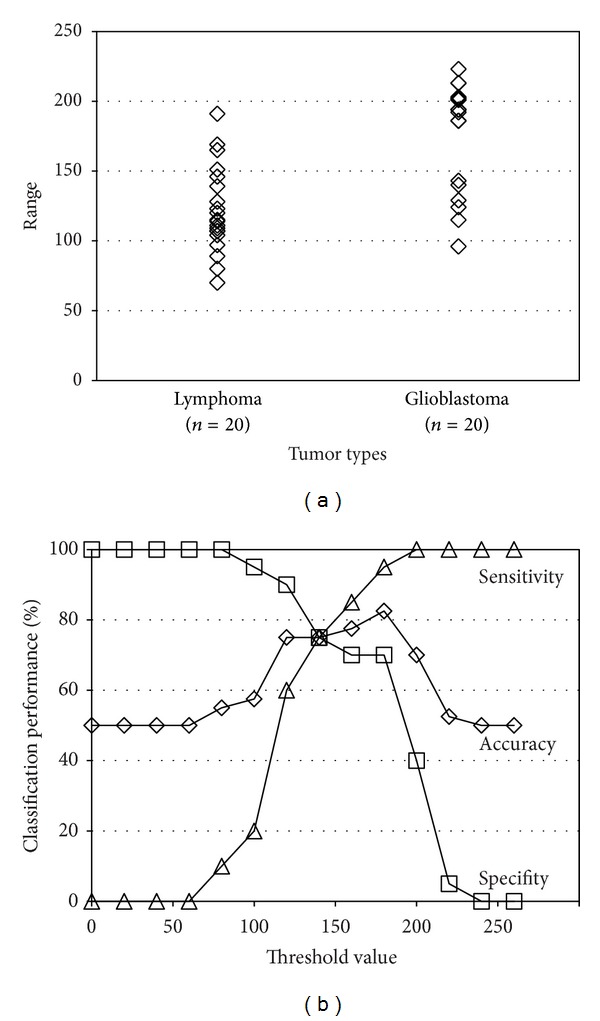
(a) Distribution of the luminance range values. (b) Classification performance when range thresholding is used. In the sensitivity/specifity calculation, lymphomas were considered as “positive” and glioblastomas were considered as “negative.”

The distribution of the luminance range values and classification accuracy by simple thresholding are shown in Figure 6. It is shown that the luminance range thresholding can classify lymphomas and glioblastomas better than the ADC thresholding [1]. The best classification accuracy is 82.5%. However, this is the result of fine tuning of the two parameters: the threshold value for the range extraction and the threshold value for classification (luminance range thresholding). The classification performance is sensitive to these threshold values and therefore this approach is not practical.

### 3.3. Classification Performance of Our Proposed Algorithm

In the experiments in this subsection, *k*(*k* = 1–19) samples per class were randomly selected for the training and the rest were used for the testing. The histograms of the typical tumors were also included in the training. This procedure was repeated 1,000 times and the average performance is presented. This random-sampling-based evaluation was employed because the threshold values depend on the training samples.

**Figure 7:**
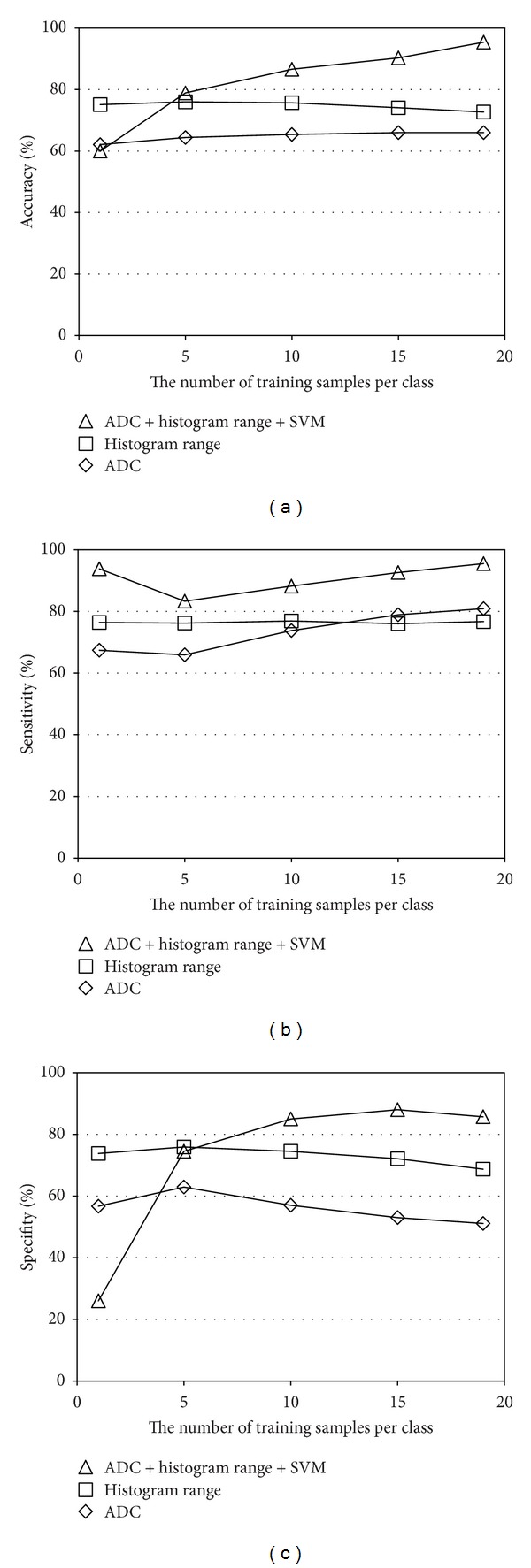
Classification performance of the proposed method and the two thresholding-based methods: (a) accuracy, (b) sensitivity, and (c) specifity.

The classification performance of our proposed method is shown in Figure 7 and it is compared with those by naïve ADC thresholding and luminance range thresholding. When the luminance distribution learning using SVM is not used, nontypical (or unknown) cases cannot be handled. Therefore, the threshold values for the ADC thresholding (TH_ADC_) and the luminance range thresholding (TH_range_) were set as follows:

(1) $$\begin{array}{c} {\text{TH}}_{\text{ADC}}=\frac{{\text{TH}}_{\text{ADC}\_\text{gli}\_\min}+{\text{TH}}_{\text{ADC}\_\text{lym}\_\max}}{2},\\ {\text{TH}}_{\text{range}}=\frac{{\text{TH}}_{\text{range}\_\text{gli}\_\min}+{\text{TH}}_{\text{range}\_\text{lym}\_\max}}{2}. \end{array}$$

It is observed that the classification accuracy of the ADC thresholding is up to 66% and that of the luminance range thresholding is 72%–76%. On the other hand, the proposed method achieves 95.4% when *k* = 19. Besides, our proposed method outperforms the other two methods when *k* is equal to or larger than 5.

**Figure 8:**
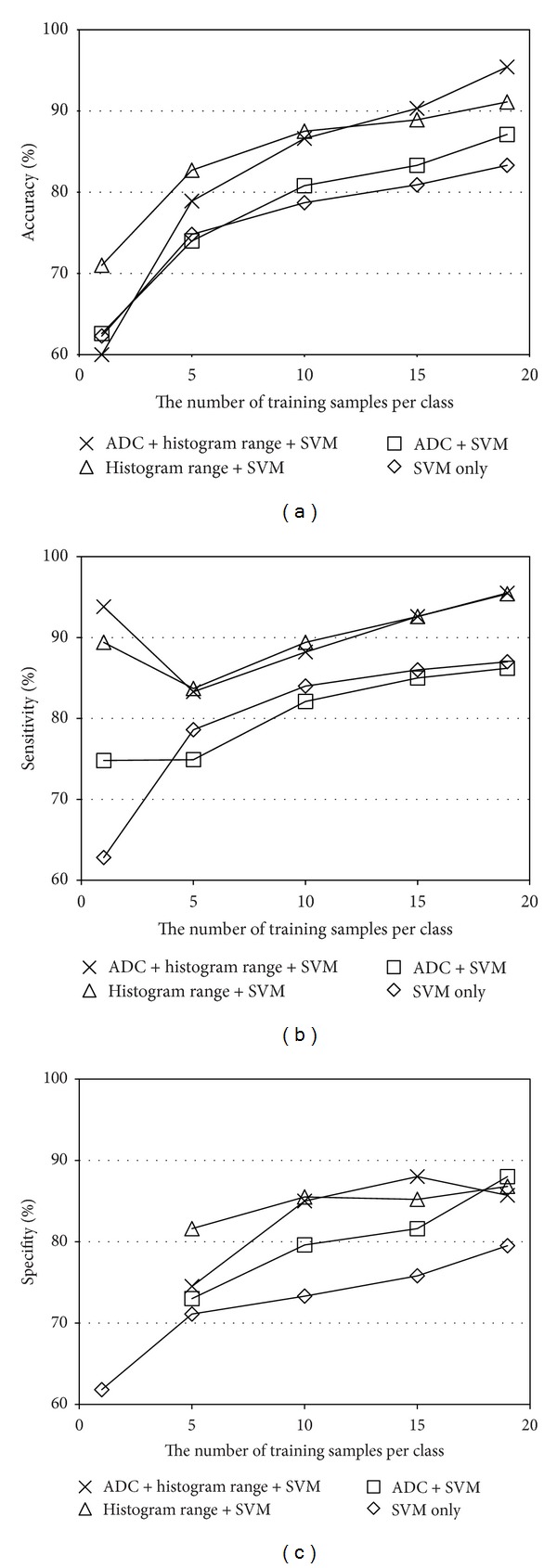
Classification performance comparison between the subsets of the proposed system: (a) accuracy, (b) sensitivity, and (c) specifity.

Figure 8 shows the classification performance when the subset of our proposed method is used. It is interesting to note that even the SVM-only approach (i.e., only the SVM trained with the luminance histograms was used) can achieve 83.3% accuracy when *k* = 19. This is significantly better than the ADC thresholding [1]. The luminance range thresholding + SVM method yields 91.1% accuracy. Since this method does not require any experiences or knowledge of the tumors, no interobserver variance exists and even novice users can get the same performance. It is also observed that the luminance range thresholding outperforms the other approaches when the training samples are less. The best performance (95.4%) was achieved when all the features are used. Namely, the typical cases were classified by using both the ADC thresholding and the luminance range thresholding and the nontypical cases were classified by the SVM using the luminance histogram. The performance of the ADC thresholding + SVM [9] was 87.1%.

**Figure 9:**
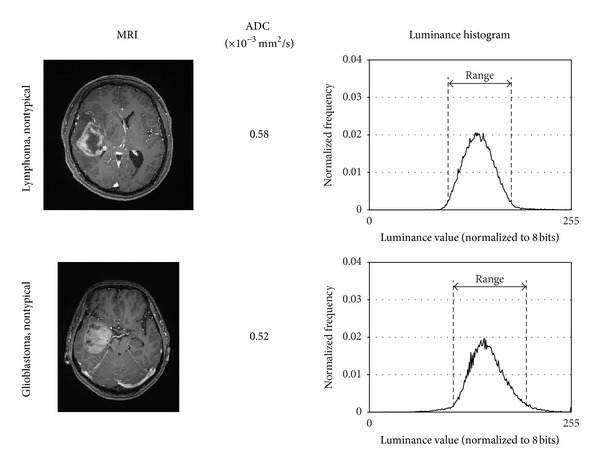
Examples of misclassified cases.

The example images which were misclassified are shown in Figure 9. The neuroradiologist could not classify them correctly just by looking at the images and ADC values. Therefore, these mistakes are reasonable and show the limitation of our proposed method.

**Figure 10:**
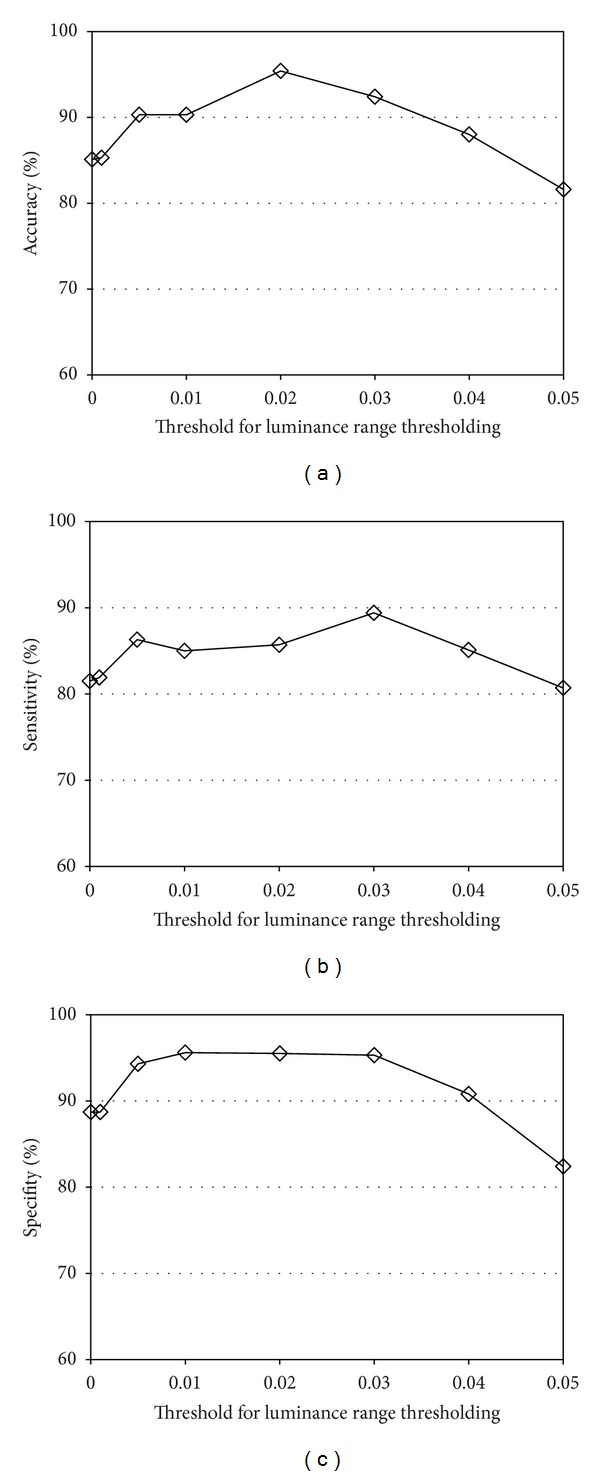
Classification performance as a function of the luminance range threshold: (a) accuracy, (b) sensitivity, and (c) specifity.

The dependence on the luminance range threshold is demonstrated in Figure 10. The best classification performance is observed at th = 0.002. As the threshold value shifts from its optimal value, the classification performance is degraded gradually. However, the classification accuracy is over 90% even when th = 0.001 or th = 0.003, showing the robustness of the proposed method.

**Figure 11:**
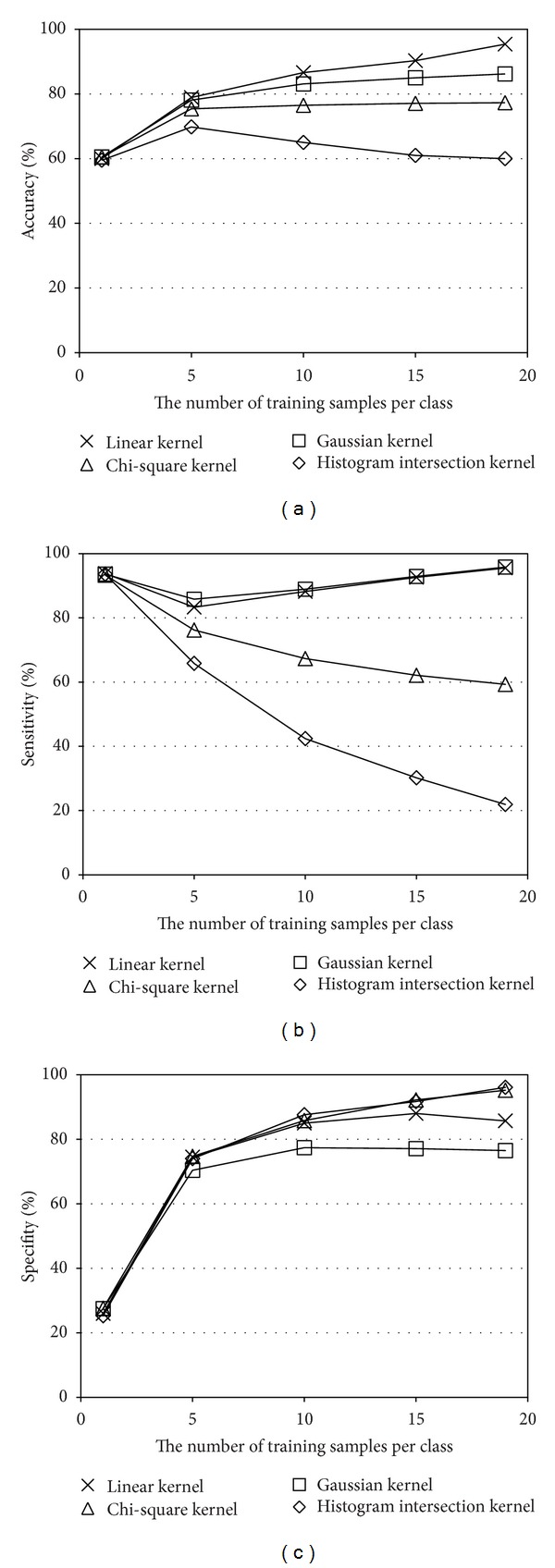
Performance comparison between four kernels: (a) accuracy, (b) sensitivity, and (c) specifity.

It is well known that SVMs using a Chi-square kernel or histogram intersection kernel work better than Gaussian or linear kernel when the feature vector is histogram based. Therefore, the four kernels are compared in Figure 11. In our framework, the linear kernel was the best. It should also be noted that the linear kernel was the fastest in training.

## 4. Conclusions

Luminance distribution analysis using the whole tumor region has been developed for differentiating lymphomas and glioblastomas. The typical tumors were classified by the luminance range thresholding. And the nontypical cases were learned and classified by SVM using the luminance histogram. Since subjective measurement of the parameters was not needed, the system was easy to use even for novice users and there was no interobserver variance. The combination of the luminance range thresholding and the SVM-based classification achieved 91.1% accuracy. Besides, when the ADC value measured by an expert was added in the thresholding, the accuracy was improved up to 95.4%.
